# Appropriate Support for “Specified Expectant Mothers”

**DOI:** 10.31662/jmaj.2021-0165

**Published:** 2021-12-08

**Authors:** Shunji Suzuki

**Affiliations:** 1 Department of Obstetrics and Gynecology, Japanese Red Cross Katsushika Maternity Hospital, Tokyo, Japan

**Keywords:** appropriate support, assessment sheet, specified expectant mothers, Japan

## Abstract

“Specified expectant mothers” are defined as pregnant women at high risk of needing extra support after birth. To provide them appropriate support, the methods for evaluating specified expectant mothers should be standardized. Thus, in this study, I reviewed the evaluation and multidisciplinary collaboration in some regions reported to be actively supporting specified expectant mothers in Japan. The main items related to “specified expectant mothers” were as follows: (1) mental disorders, (2) younger age, (3) no consultation/late first visit, (4) poverty, and (5) multiple pregnancy. It is important to proactively identify and confirm the problems faced by pregnant women through screening and interviews conducted by the medical staff.

## Introduction

In 2010, “specified expectant mothers” have been defined by the Japanese Ministry of Health, Labour and Welfare as pregnant women at high risk of abuse and/or in need of extra support after birth because of some social problems such as unstable income, mental disorders, etc ^(1)^. For example, in our institute, which is one of the main Japanese perinatal centers (about 1,700 deliveries recorded per year) located in the downtown area of Tokyo, at least 200 specified expectant mothers are managed per year ^(2)^. In our institute, “specified expectant mothers” are recognized according to the criteria devised by myself to support their social problems through multidisciplinary collaboration including regional administrative staff. In our earlier study ^(3)^, for example, to quantitatively clarify the mental vulnerabilities of “specified expectant mothers,” they were observed at 1 month after delivery using the Japanese version of the Mother-to-Infant Bonding Scale and the Edinburgh Postnatal Depression Scale.

In most obstetric institutions in Japan, pregnant women are often asked by regional administrative staff about their living environment using individual evaluation sheets at their first visits ^(4)^. On the other hand, in most municipalities in Japan, most women visiting to register for their pregnancies are screened by midwives or nurses for their social problems using an individual screening/assessment sheet ^(5)^. However, the objective methods of assessment and/or criteria for their problems have not been set clearly at many municipalities and institutions ^(4), (5)^.

To provide appropriate support to specified pregnant women, the methods for evaluation should thus be standardized. Therefore, we reviewed the actual situation of the evaluation and multidisciplinary collaboration in some regions reported to be actively supporting these group of pregnant women in Japan.

This study protocol was approved by the Ethics Committee of the Japanese Red Cross Katsushika Maternity Hospital (K2021-18). Informed consent concerning retrospective analyses was obtained from all subjects.

## Screening Tools for “Specified Expectant Mothers”

First, we examined the common items used in the screening sheets throughout Japan to determine and identify “specified expectant mothers.” Based on the status of some domestic workshops and/or academic conference reports investigated in the first and second editions of the ‘Perinatal Mental Health Care Manual’ published by the Japan Association of Obstetricians and Gynecologists ^(6), (7), (8)^, seven screening sheets created by five areas/institutes, other than our department and the Ministry of Health, Labour and Welfare, were included for examination. We have confirmed that these five areas/institutes are actively working to support specified expectant mothers, as we searched the terms “specified expectant mothers” and “assessment or support” in the Japan Medical Abstracts Society (https://search.jamas.or.jp/).

Table 1 summarizes the seven screening sheets used for “specified expectant mothers.” The number of items to be checked was 6 to 26, which was different for each sheet. The following items have been listed in at least six of the seven sheets: (1) mental disorders, (2) younger age, (3) no consultation/late first visit, (4) poverty, and (5) multiple (twin) pregnancy.

**Table 1.** Items to Extract “Specified Expectant Mothers” in the Screening Sheets Used in Eight Institutes in Japan.

	Area where the institute is located							
Associated factors for “specified expectant mothers”	Japan	North-Tokyo	South-Tokyo	Osaka	Fukuoka	Oita	Okinawa
Mental disorders	○	○	○	○	○	○	○
Physical diseases	○	○		○	○		○
Sensitivity		○					○
Extreme thinking		○					○
Difficulty in communication		○					○
Aggressive personality		○					○
History of abuse	○	○		○	○		○
History of being abused	○	○		○	○		○
Anxiety	○	○			○		○
Anorexia		○					○
Insomnia	○	○					○
Prolific		○					○
Younger age	○	○	○	○	○	○	○
Older age			○	○	○		
Single	○	○			○		○
Desire for career advancement		○			○		○
No consultation/late first visit	○	○		○	○	○	○
Poverty	○	○		○	○	○	○
Lack of support	○	○			○		○
Family requiring nursing care/medical treatment	○	○		○	○		○
Unhoped pregnancy		○		○	○	○	○
No notification		○		○			○
Multiple (twin) pregnancy	○	○	○	○	○	○	
Fetal anomaly	○	○	○	○	○		
In vitro fertilization		○	○				
Screening of bonding	○	○	○				
Screening for depression	○	○	○				

○：item listed in the sheet.

The first four factors are examined as serious risk factors for inappropriate childcare from inside and/or outside Japan ^(1), (2), (9), (10), (11), (12), (13)^. These factors have been observed to be more complex and are likely to occur in duplicate than they occur alone ^(14)^. They may be urge to receive not only medical and psychological care but also age-specific care. On the other hand, child-rearing difficulty of multiple (twins) pregnancy has been pointed out ^(15)^.

## First Support for “Specified Expectant Mothers”

To date, in our institute, we have supported their problems mainly through our multidisciplinary collaboration composed of midwives, clinical psychologists, medical social workers, medical treasurers, and regional administrative staff ^(2), (13), (16)^. For example, clinical psychologists evaluate the mental status and give advice on how to get referred to psychiatrists and/or regional staff. The medical social workers provide support and information on social resources available and serve as contact points for regional organizations. The information as regards this group of pregnant women is then shared, wherein opinions are exchanged from each specialized area, and support methods are decided.

Hereafter, the outlines of the specific support for each problem based on literature considerations are put forward.

### 1) Mental disorders

Recently, perinatal mental disorders have become a significant complication of pregnancy, in particular in the postpartum period ^(17)^. Perinatal mental disorders impair a woman’s function and are associated with the suboptimal development of her children. Perinatal mental healthcare is thus required for the emotional well-being of a pregnant woman and her children, partner, and family members. The early detection and effective management of perinatal mental disorders are critical for the welfare of a woman and her children ^(18)^. For example, women who receive appropriate psychosocial or psychological intervention have been observed to be significantly less likely to develop postpartum depression compared with those receiving standard care ^(18)^. There may be a number of steps in perinatal psychosocial or psychological intervention that help a pregnant woman to get her emotions in check during pregnancy and after childbirth. Promising interventions include the provision of intensive, professionally based postpartum home visits, telephone-based peer support, and interpersonal psychotherapy.

Recently, some clinical guides for women with mental health problems during the perinatal period have been published to facilitate healthy pregnancies and childbirth ^(7), (19), (20)^. Based on the guidelines, it should be noted that a woman’s physical conditions change significantly during pregnancy due to changes in maternal circulating blood volume and hormones; moreover, use of pharmaceutical products among pregnant women should be prohibited as it can affect the child, depending on the stage of pregnancy ^(19)^. Clinical evidence on the safety of psychotropic medicines during the perinatal period remains limited; however, the incidence of deterioration/relapse of mental disorders has been noted to be higher in women who discontinued their medications for their mental health problem regardless of doctor’s discretion or self-interruption compared to women who continued their medications during pregnancy. Therefore, the continuation of medications and careful observation should be required for pregnant women complicated by mental disorders ^(21)^.

In recent years, some insurance listings and subsidies have been newly established on the premise of medical cooperation between obstetricians and psychiatrists and/or support by multidisciplinary cooperation. Moreover, the mental healthcare of pregnant women in obstetric facilities has become a public demand ^(7), (22)^. These are based on the awareness that the government, which had been waiting for consultations and support requests from pregnant women, can actively reach out to pregnant women who need support. In particular, the prevalence of perinatal depression has been observed to be as high as 10-15 %, and it may be unrealistic to refer all of them to psychiatry. Most perinatal depression seems to be mild and is associated with some obvious social factors during pregnancy and postpartum, and it will be improved by solving them ^(7), (22)^. Considering the long-term support, it will be desirable for them to be managed in an accessible maternity facility near their home.

### 2) Younger age

Some previous studies have reported that pregnancy at younger age is a risk factor for premature delivery and neonatal hospitalization; ^(23)^ however, almost all observations have suggested its low medical risk ^(16), (24), (25)^.

In our previous studies ^(16), (24)^, there were some cases with first perinatal visit after 22 weeks’ gestation, unknown partners, and/or economic problems in younger aged pregnancy. In Japan, when a minor gives birth without marriage, the parental authority of the neonate is legally required by the parents of the woman. Therefore, it is important to have a relationship between the younger aged women and their parents for pregnancy and childcare. However, in some cases, the relationships are not good, and we sometimes cannot even contact their parents. It is feared that delaying consultation due to economic problems and/or being unable to consult with their families while on pregnancy may lead to delays in required medical and/or social supports.

In addition, although there were some cases of marriage with their partners after delivery, the divorce rate in women under the age of 19 has been reported to be as high as 60 % in Japan ^(26)^. If young women without special skills become child caregivers alone, this will only add further distress on their lives. Therefore, independent financial and social support coming from welfare offices, visits and telephone guidance by public health nurses, and group support by public health centers and/or health centers is needed for young aged pregnancy, especially after delivery.

### 3) No consultation/late first visit

The increased number of pregnant women without prenatal care has become a serious problem in Japan. The unpreparedness of women for their upcoming deliveries does not only affect themselves but also their fetuses. The situation also may create a huge burden for the obstetric institutes.

The main reasons leading to no prenatal visit have been reported to be as follows: economic problems, unaware of pregnancy, unable to consult with anyone, and due to being busy. Recently, cases with complex home environment (unaware of pregnancy and having no one to consult with) have been noted to increase ^(27)^. In these cases, it is necessary to identify the real problems in these situations and take measures to prevent their social isolation. Recent changes in family forms and communication methods such as social networking service (SNS) might have contributed to the isolated situation of a pregnant woman. Thus, various social support using SNS should be publicized to all non-pregnant and pregnant women. In addition, “being busy” and “ignoring pregnancy” may suggest the lacking awareness on the possible risks of pregnancy and delivery. Therefore, the support required by women without prenatal care may be the improvement of the negative effects of a complex home environment rather than economic support.

### 4) Poverty

In Japan, the hospitalization assistance policy (HAP) system, based on the Child Welfare Act, has already assisted a number of pregnant women who cannot give birth at medical institutions for economic reasons ^(28)^. In this HAP system, women were allowed to deliver at specified (midwifery) institutions. The main objective of this system is to help pregnant women receive livelihood protection, as they are unable to maintain minimum living standards because of poverty, live in households exempt from the residence tax, and live in households in which the income tax is less than about 100 US dollars per year.

However, unfortunately, there have been some underage and/or non-Japanese women who are not aware of this system or specified midwifery institutions ^(29)^. They were usually refused medical examinations and treatments at some non-specified midwifery institutions for economic reasons without being informed of the HAP system.

However, women who actually need social supports are those with minimum income that does not meet the subsidy criteria of the HAP system. Without financial support, pregnant women will not be able to afford quality healthcare they deserve. These are often pregnant women who have escaped the violence of their earning husbands. We sometimes have offered installments of their delivery costs; however, it will not be possible at all facilities.

### 5) Multiple (twin) pregnancy

Some mothers with multiple (twins) pregnancy have been pointed out to have a strong sense of child-rearing difficulty associated with delayed childhood development and be mentally cornered with respect to child-rearing ^(15)^. In an earlier study by Yokoyama et al. ^(30)^, multiples and their mothers had a higher rate of risk factors for child maltreatment such as low birth weight and neural abnormality. In addition, compared with mothers of singleton, mothers of twins are often more prone to developing poor health. Recent Japanese nationwide data have suggested that the non-specific overburden of child-rearing might be one possible reason for higher frequency of child maltreatment for multiples compared with singletons, and parental comparisons between two twins might be another ^(31)^. In cases of twins with a considerable difference in birth weights, it has been reported that stunted infants tend to be the target of abuse ^(32)^.

Parenting multiples is deemed very a difficult task; however, appropriate support to these mothers has remained scarce. Mothers who carry out multiple births have been gaining attention from society every time an abuse case occurs, thus revealing the difficult situation of parenting multiples. However, further elucidation of the fact that difficult childcare is not limited to multiple births has not been widespread ^(33), (34)^.

For parenting multiples, it is important to have family members who understand the difficulty of raising multiple children and lend their help when needed ^(33)^. Moreover, support from midwives and public health nurses during pregnancy and postpartum has been also suggested to be indispensable. In addition, having a circle of members who also experienced multiple births has been found to be really encouraging for them. However, there are now regional disparities in support for multiple births depending on the presence or absence of a regional multiple birth network and/or circle.

## Discussion

In this study, we reviewed the main risk factors requiring social supports assumed in multiple regions; however, there have not been any criteria for how many items should be considered as high risk. These risks have been inconsistent in different parts of Japan ^(4), (35), (36)^. They also have depended on the environments in which the woman was raised. Examining the current situation nationwide, however, there are some areas where the hurdles for designation as “specified expectant mothers” are very high. Although they have been only expected with child-rearing difficulties after birth, they may be misunderstood as they are designated as “preliminary women for child abuse” ^(37)^. We understand that fetuses of “specified expectant mothers” may be affected by epigenetic factors, but we cannot deny the possibility that early postnatal growth environment can significantly affect subsequent growth.

Therefore, appropriate support necessary for the mother should be considered if she has even one risk factor.

## Conclusions

First of all, it is important to earn the trust of a pregnant woman and their family so that they can express their feelings honestly and inform us actively when there is danger in terms of raising their children.

For women, pregnancy may be an opportunity to recognize the potential problems women have, review their lifestyles, and receive medical treatment for long life vision. To determine “what should be done for the health of the mother and their children,” it is important to proactively confirm the problems faced by a pregnant woman via screening and interviews conducted by the medical staff, especially for “specified expectant mothers.”

## Article Information

### Conflicts of Interest

None

### Author Contributions

Shunji Suzuki: all project development, data management, data analysis, manuscript writing/editing.

### Approval by Institutional Review Board (IRB)

The study protocol was approved by the Ethics Committee of the Japanese Red Cross Katsushika Maternity Hospital (K2021-18).

### Informed Consent

Patients’ informed consent for publication of this report was obtained
